# Eicosanoid-immunometabotypes reveal genotypic links to cervicovaginal pH in progestagen synchronised South African Dohne Merino ewes

**DOI:** 10.3389/fvets.2026.1851840

**Published:** 2026-07-21

**Authors:** Rimbilana Shingange, Edward Cottington Webb, Madelien Wooding, Carina Visser

**Affiliations:** 1 Department of Animal Science, Faculty of Natural and Agricultural Sciences, University of Pretoria, Pretoria, South Africa; 2 Department of Chemistry, Faculty of Natural and Agricultural Sciences, University of Pretoria, Pretoria, South Africa

**Keywords:** assisted reproductive technology, dysbiosis, genomics, GWAS, intravaginal pessary, metabolomics, PGF_2α_

## Abstract

Metabolites can be detected in various bodily fluids such as blood, urine and cervicovaginal secretions. Cervicovaginal fluid has distinct characteristics such as colour and pH that are affected by the underlying health of an ewe and her associated metabolic and endocrine processes dedicated towards maintaining homeostasis particularly after stress is experienced. This study profiled distinct metabolomic phenotypes defined by the pattern and abundance of eicosanoid mediators that reflect the underlying state of Dohne Merino ewes’ immune activation and metabolic regulation based on their comparative cervicovaginal metabolome and pH before and after the administration of an intravaginal pessary using high resolution liquid chromatography mass spectrometry. Further, single nucleotide polymorphisms (SNPs) were analysed using genome-wide association analysis (GWAS) to correlate genomic effects of cervicovaginal pH. Twenty ewes were allocated to each treatment group defined by a CIDR-, sponge- or injection-based estrus synchronisation protocol and their cervicovaginal fluid sampled using FLOQSwabs. This study matched three features to metabolites of the KEGG *Ovis aries* eicosanoid synthesis and breakdown pathway and found a significant effect of sampling point, prior to and after pessary administration, on cervicovaginal pH (*p* < 0.05). Sponge-based estrus synchronisation significantly increased cervicovaginal pH compared to the CIDR-based group (*p* < 0.05) but not compared to the control group (*p* > 0.05), where CIDR-based and control group ewes did not differ significantly from each other (*p* > 0.05). Ten SNPs were correlated to cervicovaginal pH with GWAS results visualised using Manhattan, QQ, principal component analysis and volcano plots where effect sizes (*β*) ranged from −0.5 to 1.5, minor allele frequencies (MAF) from 0.23 to 0.49 and the genomic inflation factor *λ* was 1.053. SNPSs were mapped to genes regulating mucosal inflammatory signalling, epithelial integrity, and metabolic stress responses, offering a biologically plausible mechanism through which genetic variation interacts with device-induced disruptions to cervicovaginal homeostasis to produce significantly higher vaginal pH in sponge-treated ewes. This study advocates for the continued robust characterisation of metabotypes for phenotypes relevant to livestock production and advances the conceptual framework of immunometabolism by incorporating pH as a critical axis of regulation within reproductive mucosal environments of ewes.

## 1 Introduction

The cervicovaginal microenvironment plays a pivotal role in reproductive health of female mammals and serves as a dynamic interface between host immunity and microbial communities. In sheep, the estrous cycle causes profound physiological and biochemical changes within this niche that influences both immune responses and metabolic profiles. For example, increases in oestrogen during estrus promotes the secretion of less viscous and more abundant vaginal secretions (1, 2). Estrus synchronisation, a common practise in livestock reproductive management, offers a controlled framework to investigate these cyclical alterations and their implications for disease susceptibility and reproductive success. Additionally, recent advances in metabolomics have enabled the identification of novel phenotypes that increase scientific understanding of underlying biological states. Analytical techniques such as liquid-chromatography coupled to high-resolution mass spectrometry (HR-LCMS) allow sensitive, parallel quantification and qualification of metabolites from easy-to-collect samples such as milk and blood (3, 4).

Amongst the critical factors affecting immune-metabolic interactions is pH, which can influence the protonation state of metabolites, thereby affecting their bioactivity and signalling capacity. This proton-dependent modulation of metabolism and immunity gives rise to what can be termed eicosanoid-immunometabotypes: distinct cervicovaginal metabolomic phenotypes that are characterised by coordinated variation in eicosanoid mediators, particularly prostaglandin F₂*α* (PGF_2α_), reflecting differences in ewe inflammatory and metabolomic responses. These phenotypes were derived using multivariate analyses of metabolomic data, including principal component analysis and clustering approaches, enabling the identification of structured variation in metabolite abundance profiles across treatment groups.

Understanding these immunometabotypes in the cervicovaginal fluid of sheep post-estrus synchronisation may elucidate mechanisms governing mucosal immunity, microbial homeostasis, and reproductive outcomes. Importantly, the immunity of body systems is dependent on eicosanoids. Eicosanoids, including prostaglandins, leukotrienes and thromboxanes are crucial regulators of inflammatory response in livestock (4). This response partially determines the effectiveness of disease resistance, wound healing and the efficiency of reproductive cascades through energy allocation towards hormone synthesis and histological cell health. Further, this response will be at least partially determined by the underlying genotype of the animal.

Estrus synchronisation protocols very often prescribe the use of intravaginal pessaries, generally CIDRs or sponges, that are placed in the vaginal canal and release progesterone for the treatment duration, ranging from 8 to 14 days (5, 6). The impact of estrus synchronisation in general and specifically intravaginal pessary protocols has been an emerging point in recent livestock research. Authors have demonstrated that these pessaries have the potential to alter the cervicovaginal microbiome and trigger inflammatory response at the expense of reproductive performance (6–8).

Inflammation will primarily be mitigated by eicosanoids, bioactive lipids or fatty acid derivatives that are products of arachidonic acid metabolism (4). Arachidonic acid is a 20-carbon polyunsaturated fatty acid found mostly in animal tissues and is the primary substrate for the synthesis of eicosanoids through cyclooxygenase (COX), lipoxygenase (LOX) and cytochrome P450 pathways (9). Eicosanoids, through their influence on a plethora of biological functions including cell proliferation and migration and inflammatory responses, impacts livestock survival, growth and reproduction (10, 11). The quantification of eicosanoids is thus an important step in the amalgamation of animal welfare and technologies aimed at improving livestock production parameters (12).

This study’s aim was to, firstly, detect and annotate eicosanoids from cervicovaginal fluid prior to and immediately after intravaginal pessary estrus synchronisation. Secondly, to correlate these eicosanoids to the pH of cervicovaginal fluid to generate viable cervicovaginal eicosanoid-immunometabotypes in sheep following estrus synchronisation. Lastly, to use a genome wide association (GWAS) to detect single nucleotide polymorphisms (SNPs) and associated genes that may contribute to the immunometabotypes observed. The null hypothesis was that there would be no significant difference in cervicovaginal pH or target metabolite abundance between treatment groups, with no GWAS SNPs being correlated to cervicovaginal pH.

## 2 Materials and methods

### 2.1 Ethical approval

Ethical approval was obtained from the University of Pretoria’s Animal Ethics Committee (NAS 162/2022) as well as a Section 20 permit from the South African Department of Agriculture, Land Reform and Rural Development (12/11/12777KL).

### 2.2 Study area

This study was conducted at the University of Pretoria’s Innovation Africa farm in South Africa in June, which is the natural breeding season, short days, of Dohne Merino sheep in the southern hemisphere (13, 14).

### 2.3 Animals and their management

The chosen flock is a dedicated research flock in South Africa, where the Dohne Merino breed is one of the most numerous and thus commercially relevant (15). Ewes were all kept in the same pen and grazing-group over the period of the study, with *ad-lib* access to water and grazing on kikuyu (*Cenchrus clandestinus*) grass.

### 2.4 Estrus synchronisation

This study was an experimental quantitative trial (*n* = 60) with three parallel groups, in which ewes were assigned to three treatments based on weight and parity. The CIDR-based treatment group had a mean weight of 55.24 kg; the Sponge-based treatment group, 55.5 kg; and the non-pessary treatment group, which was considered the control group, 54.79 kg. All groups had 30% nulliparous ewes, 10% primiparous, and 60% biparous to pentaparous ewes. The non-nulliparous ewes had an average of 82.96 ± 12.99 days since their most recent partition, sufficient time for the reversion of metabolic state and uterine histology to pre-partum and pre-gestational conditions (16, 17). None of the ewes had been previously estrus synchronised by any protocol.

After the estrus synchronisation protocol used that is tabled in Table 1, estrus response was assessed at laparoscopic insemination by 48 h later by detection of a dominant follicle, assessment of firmness of uterine horn, swollen vulva and mucus discharge (18).

**Table 1:** Experimental design and estrus synchronisation treatment groups.

Treatment group	CIDR-treated	Sponge-treated	Control
Sample size	20	20	20
Day 0	15 mL OviThrive (Ovi-Thrive Sheep and Goats with Copper, Ashkan Consulting (Pty) Ltd) (oral). 1 mL/50 kg melatonin (subcutaneous).		
Day 16 (*Immediately prior to treatment*)	CIDR (EAZI-BREED™ CIDR^®^ Sheep Insert, Zoetis) insertion	Sponge (GenoCo, South Africa) insertion	2 mL Estrumate (Estrumate^®^, Merck Animal Health) (intramuscular)
Day 24 (*Immediately post-treatment*)	CIDR removal. 2.5 mL Chronogest (Chronogest^®^ PMSG 6000 IU, Merck Animal Health) (intramuscular).	Sponge removal. 2.5 mL Chronogest (intramuscular).	2 mL Estrumate (intramuscular). 2.5 mL Chronogest (intramuscular).

### 2.5 Cervicovaginal pH and metabolome

Before the treatments listed in Table 1 were administered, cervicovaginal fluid samples were collected from all ewes (*n* = 60), regardless of treatment group. These samples were considered as homeostatic metabolomes. For all sampling, an unused sterile, packaged speculum was used for each ewe, where an 80 mm FLOQSwab (Copan, Brescia, Italy) were used for transvaginal sampling before removal of the speculum. The ewe’s perineum was clean while securely restrained, and the swab inserted along the long axis of the vaginal canal until the posterior cervix where equal rotations clockwise and counterclockwise were made to sample the cervix and vagina as the swab was retracted.

pH was determined via modified protocols using a Thermo Scientific pH metre with glass electrode (19, 20) and a steroid internal standard mix was purchased from Microsep (Waters™, Microsep, South Africa) as the pure chemical standard for metabolomics.

Details of metabolite extraction and instrument method are provided in Supplementary materials. Resultant multivariate feature tables after metabolite extraction using high resolution liquid chromatography mass spectrometry (HR-LCMS) were created using the UNIFI Scientific Information System (UNIFI) (Waters Inc., Milford, Massachusetts, United States) for further statistical processing. Feature table data was pre-processed and pretreated before statistical analysis as follows: Features were aligned at 10 ppm for mass to charge ratio (m/z) 0.1 min for retention times (RT) (minutes). Detected features were normalised by quantile (21) without filtering in MetaboAnalyst 6.0’s R Studio package (22, 23). A general linear model with covariate adjustments was used to perform significance testing wherein Raw *p*-values between groups per feature were generated (24).

#### 2.5.1 Quality control of samples

All cervicovaginal samples were duplicated by the successive use of two swabs both prior to treatment at Day 16 and immediately post treatment at Day 24 per ewe to minimise experimental error plausibly correlated to a specific swab. Additionally, to detect meaningful differences in biological samples and detect both enriched and depleted metabolites while removing ambiguous or noise-level signals, field blanks, solvent blanks and swab blanks were included in analysis (25).

#### 2.5.2 Target metabolites

The KEGG *Ovis aries* database lists various eicosanoid metabolites under the arachidonic acid metabolism pathway and of those metabolites, only those that have been reported to be detectable using HR-LCMS in the absence of rapid stabilisation techniques or derivatisation were considered (26–30) (see Supplementary material).

#### 2.5.3 Statistical analysis

The stat R package was used to perform ANOVA analysis on features and obtain *p*-values (31). To compare treatment group’s means, this package was used to perform Tukey’s Honestly Significant Difference (HSD) post-hoc statistical test to generate between-group differences and their significance. Similarly, this package was also used to generate group and sampling point means with standard deviations. Treatment group was blocked for to control for treatment-related variability and parity was not included as a blocking factor due to its uniform distribution across groups as per the design of experimental group.

### 2.6 Association analysis

#### 2.6.1 Sample collection and genotyping

Jugular venipuncture was used to collect peripheral blood from each ewe while securely restrained and the collection site disinfected. Blood (6 mL per ewe) was drawn into ethylenediaminetetraacetic acid (EDTA) vacutainer tubes using a sterile 21-gauge needles. Samples were gently inverted to allow mixing with anticoagulant and stored at 4 °C before being transported to the laboratory for DNA extraction and genotyping.

Samples were genotyped using the Infinium™ OvineSNP50Kv3 array (Illumina Incorporated^©^) with 63,840 SNPs according to the manufacturer’s protocol and GenCall Version 7.0.0 and a low score cutoff of 0.15 (Unistel^®^, South Africa).

#### 2.6.2 Quality control

A sample call rate of 0.02, a SNP call rate of 0.01 and minor allele frequency of 0.01 was used to discard SNPs. After linkage disequilibrium (LD) pruning using a window size of 50, a step size of 5 and r^2^ of 0.5, remaining SNPs were used to generate ten principal components as LD pruning removes correlated SNPs. Genotypes were analysed using PLINK v1.9 (32) where linear regression of cervicovaginal pH on each SNP, Treatment group, CIDR, sponge or control, was included as a fixed effect, with parity and principal components being covariates as per the below linear regression model:


$$y=\mu +\text{Treatmen}t_{i}+\text{Parit}y_{j}+\sum_{k=1}^{10}PC_{k}+\varepsilon$$

In this model, y represents cervicovaginal pH, μ is the overall mean, ${\text{Treatment}}_{i}$ is the fixed effect of treatment group (CIDR, sponge, or control), ${\text{Parity}}_{j}$ is the categorical effect of parity, ${\mathrm{PC}}_{1}$ through ${\mathrm{PC}}_{10}$ are the first ten derived principal components, and ε is the residual error.

PLINK results were visualised as a Manhattan plot, Quantile-Quantile (QQ) plot, volcano plot and PCA plots using R (version 4.4.2) for the interpretation of genetic signals and population structure (31, 33–36).

#### 2.6.3 SNP annotation and KEGG analysis

GWAS summary statistics generated in PLINK were post-processed in R using the biomaRt and data.table packages (22). The SNPs identified through LD clumping were selected for annotation. For each SNP, chromosomal position, effect size (BETA), test statistic (STAT), and raw *p*-value were extracted from the GWAS output. SNPs were annotated using the Ensembl database via biomaRt, querying the *Ovis aries* gene dataset. The nearest protein-coding gene to each SNP was identified based on genomic position, and the distance to the nearest gene start site was calculated in base pairs. Gene identifiers, gene symbols, and functional descriptions were retrieved and merged with GWAS summary statistics. Approximate 95% confidence intervals for SNP effect sizes were calculated as BETA ± 1.96 × SE, where standard errors were obtained directly from the GWAS output. Effect sizes and statistical significance were visualised by plotting BETA against −log10(*p*-value).

Candidate genes identified through Ensembl-based SNP annotation were further examined for functional relevance using the Ensembl genome browser (37) and were additionally queried against the Kyoto Encyclopaedia of Genes and Genomes (KEGG) database to identify known pathways and biological processes in which they are involved (26).

## 3 Results

### 3.1 Cervicovaginal pH

The summary statistics of cervicovaginal pH show that at Day 16 (prior to treatment), cervicovaginal values were similar across groups, with mean (± SD) values of 6.21 ± 0.400 for CIDR-treated, 6.25 ± 0.326 for sponge-treated, and 6.30 ± 0.496 for control animals. Median values followed a similar pattern (6.10, 6.21, and 6.27, respectively). Notably, at Day 24 (immediately post-treatment), the CIDR-treated group showed a lower mean value (5.89 ± 0.293; median 5.89) compared to the sponge-treated (6.29 ± 0.345; median 6.37) and control groups (6.02 ± 0.543; median 5.92), while the sponge-treated group had the highest central tendency.

The results of the ANOVA analysis of pH in Table 2 showed that cervicovaginal pH is significantly affected by sampling point, being day 16 or day 24 (*p* < 0.05). The comparatively high *F*-value supported this finding, showing that variation between groups is significantly larger than the variation within groups (*F*-Value = 6.481).

**Table 2:** The ANOVA analysis of the effect of CIDR and sponge estrus synchronisation treatments compared to a negative control group in Dohne Merino ewes prior to pessary insertion and immediately post-pessary removal on cervicovaginal pH.

Source of variation	Degrees of freedom	Sum of squares	F-value	*p*
Group	2	0.974	2.964	0.056
Sampling point	1	1.065	6.481	0.012
Group × Sampling point	2	0.802	2.440	0.0917
Error	114	18.728		

Accordingly, in terms of Tukey’s HSD pairwise group comparisons, at Day 16 (prior to treatment), there were no significant differences in cervicovaginal pH between groups. Comparisons showed small, non-significant mean differences between CIDR-treated and control ewes (−0.089; SE = 0.128; adj. *p* = 0.767), CIDR- and sponge-treated ewes (−0.039; SE = 0.128; adj. *p* = 0.950), and control versus sponge-treated ewes (0.050; SE = 0.128; adj. *p* = 0.919). However, At Day 24 (immediately post-treatment), the CIDR-treated ewes’ cervicovaginal pH was significantly lower than the sponge-treated ewes’ (mean difference = −0.402; SE = 0.128; adj. *p* = 0.006). Differences between CIDR-treated and control ewes (−0.125; SE = 0.128; adj. *p* = 0.592) and between control and sponge-treated ewes (−0.277; SE = 0.128; adj. *p* = 0.082) were not statistically significant, although the latter showed a trend toward significance.

### 3.2 GWAS

**Figure 1:**
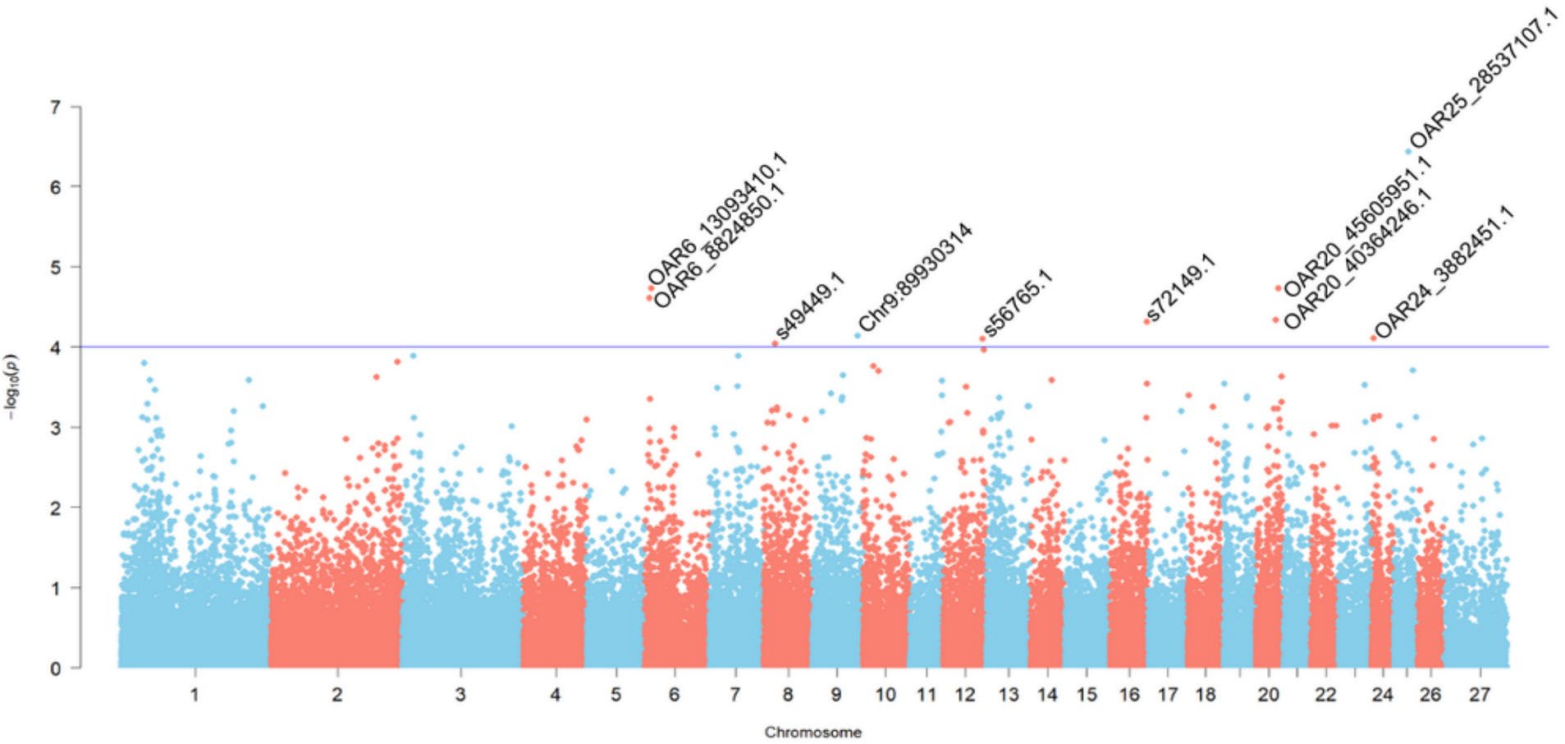
Manhattan plot of SNP associations with cervicovaginal pH of Dohne Merino ewes post-CIDR, sponge, or control treatment with parity and ten principal components as a covariate.

Figure 1 shows a Manhattan plot of the GWAS of cervicovaginal pH immediately post-treatment. This analysis revealed 10 candidate loci below a suggestive threshold of *p* < 1 × 10^−4^ commonly used in livestock SNP-chip studies and balances discovery with control of false positives (38, 39). Prior to quality control, 63,743 variants were found from the 60 ewes’ samples. After quality control, 55,826 SNPs and all 60 ewes were retained for linear regression analysis.

**Figure 2:**
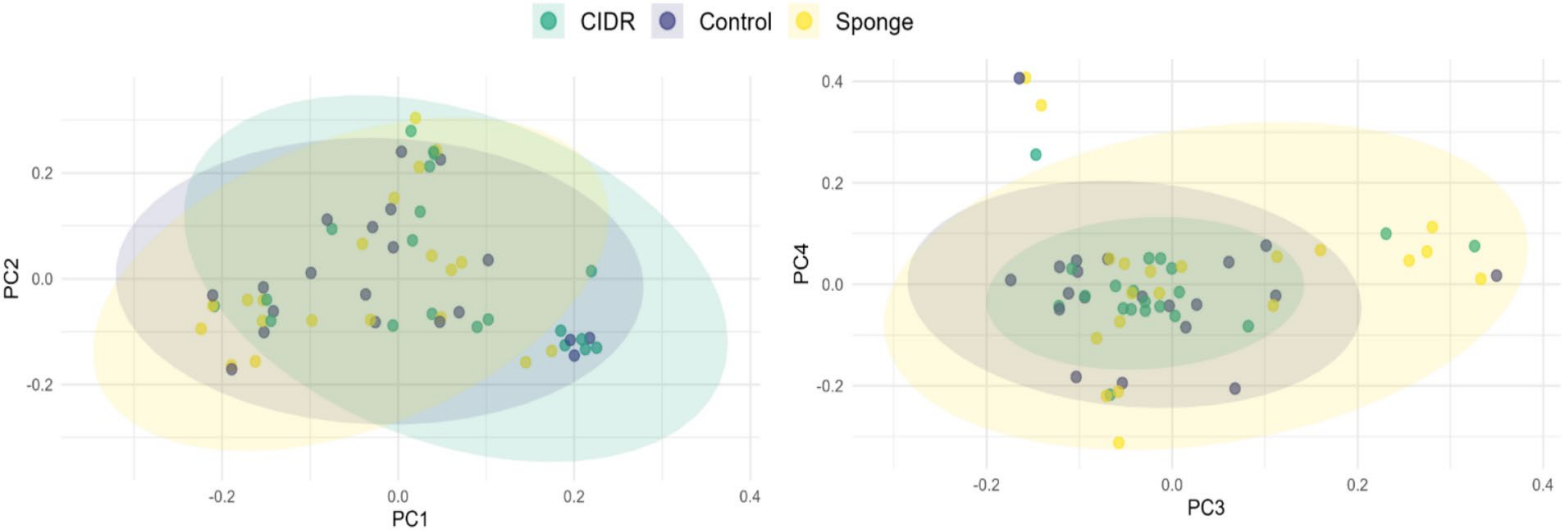
Principal component 1 vs. 2 and principal component 3 vs. 4 of Dohne Merino ewes clustered by CIDR, sponge, or control treatment group.

The principal component analysis (PCA) used to assess population structure is shown in Figure 2 and demonstrated that ewes clustered as a genetically homogeneous population, with no meaningful separation between the CIDR, sponge, and control treatment groups on either PC1 vs. PC2 or PC3 vs. PC4 axes.

**Figure 3:**
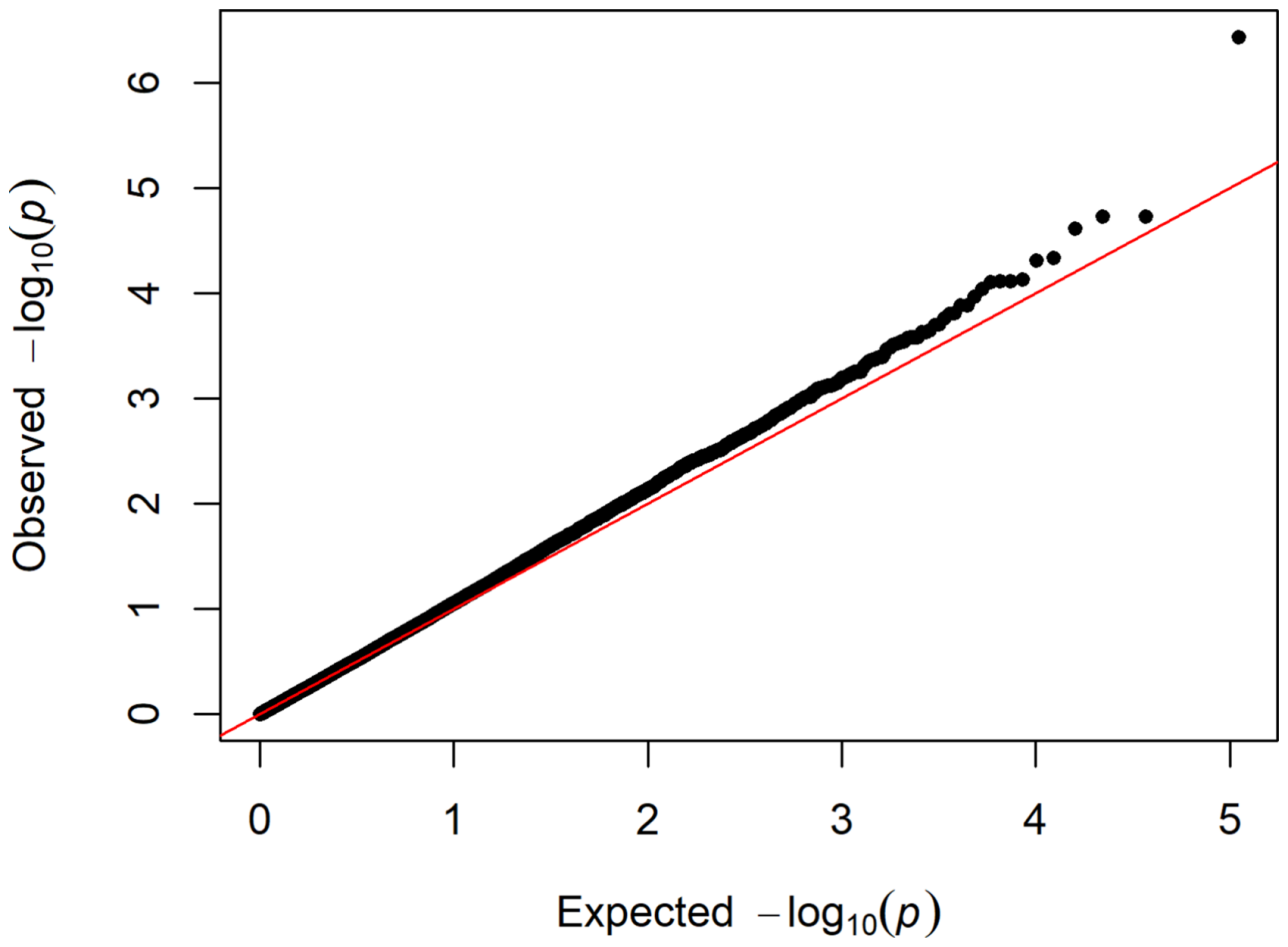
QQ plot of observed vs. expected *p*-values for cervicovaginal pH genome-wide association study of Dohne Merino ewes immediately post-CIDR, sponge, or control treatment.

The QQ plot in Figure 3 supported the above interpretation where the observed distribution of *p*-values closely followed the expected null distribution with minimal deviation. The genomic inflation factor, *λ*, was 1.053, indicating very limited inflation of test statistics (40–42).

**Figure 4:**
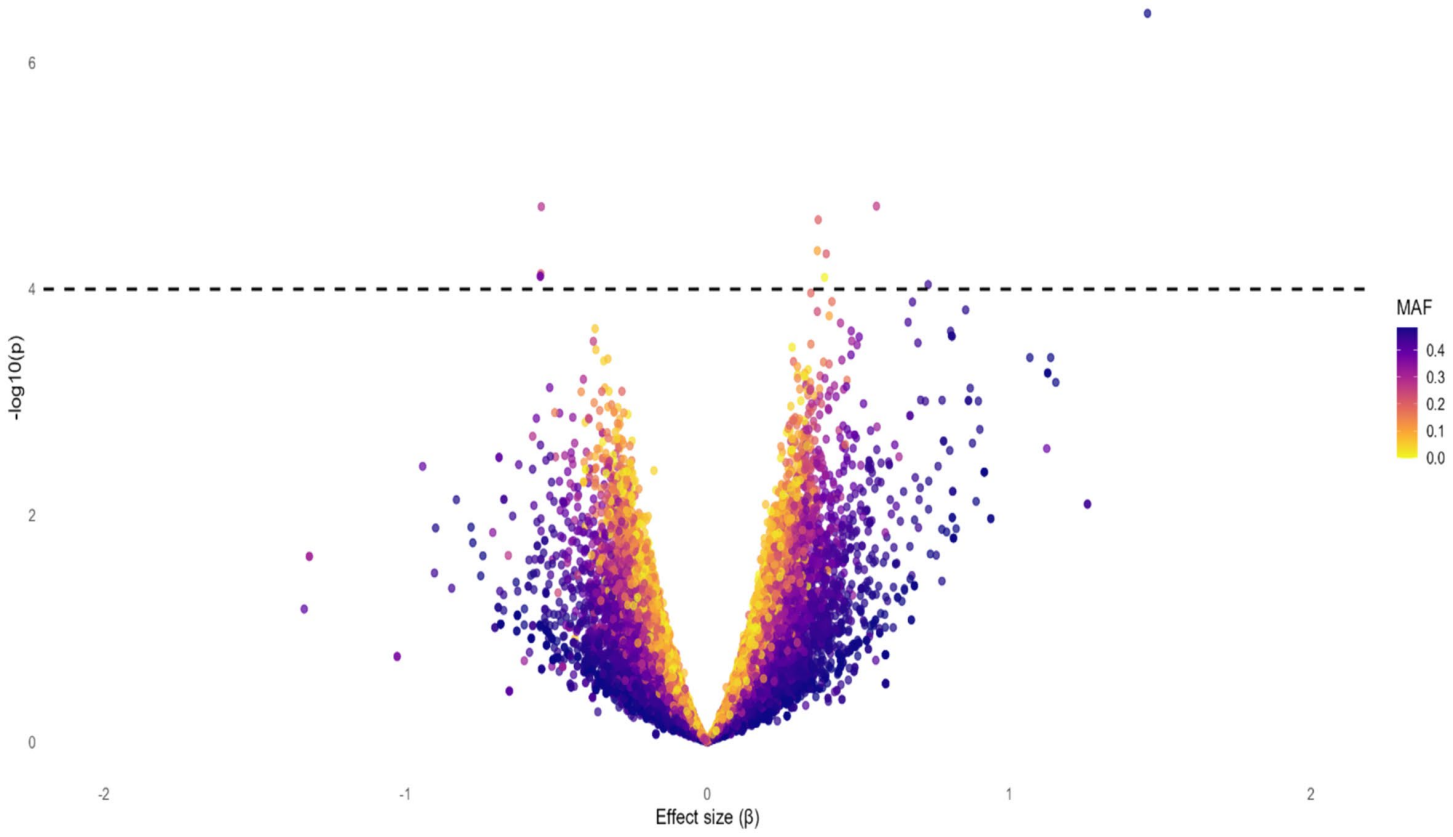
Volcano plot association of SNP effect size (*β*) and its statistical significance (−log_10_(*P*)) for cervicovaginal pH of Dohne Merino ewes immediately post-CIDR, sponge, or control treatment, coloured by minor allele frequency (MAF).

Effect sizes and statistical significance of SNPs correlated to cervicovaginal pH in Dohne Merino ewes immediately post CIDR- or sponge-treatment are integrated as a volcano plot in Figure 4. Points are coloured by minor allele frequency (MAF), with rarer alleles in darker colours and more common alleles in lighter colours. Of the 55,826 SNPs, 10 exceeded the −log10(P) = 4 threshold and represented potentially meaningful associations with cervicovaginal pH, with effect sizes (*β*) ranging from −0.5 to 1.5.

Table 3 shows the annotation information of the top SNPs associated with cervicovaginal pH, including their chromosome (OAR) and base pair location (BP); effect size (*β*); raw *p*-value (P); and nearest annotated gene and distance from that gene. The effect sizes indicate the direction and magnitude of the SNP’s influence on cervicovaginal pH, with positive *β* values corresponding to higher pH levels observed in the sponge treatment group.

**Table 3:** Annotation information of related SNPs associated with cervicovaginal pH of Dohne Merino ewes post-CIDR, sponge or control treatment with parity and principal components as covariates.

SNP	OAR	BP	MAF	*β*	*P*	Nearest gene	Distance
OAR25_28537107.1	25	28537107	0.01667	1.459	3.66E-07	Mitochondrial calcium uptake 1(*MICU1*)	271739
OAR20_45605951.1	20	45605951	0.2333	0.5605	1.85E-05	ENSOARG00000026886	197758
OAR6_13093410.1	6	13093410	0.2167	−0.5501	1.87E-05	La ribonucleoprotein 7, transcriptional regulator (*LARP7*)	20497
OAR6_8824850.1	6	8824850	0.3	0.3675	2.44E-05	14–3-3 domain-containing protein	52408
OAR20_40364246.1	20	40364246	0.4	0.3647	4.58E-05	Dystrobrevin binding protein 1 (*DTNBP1*)	23817
s72149.1	16	74648481	0.2917	0.3941	4.87E-05	ENSOARG00000027002	2936985
Chr9:89930314	9	89930314	0.2917	−0.5519	7.30E-05	Ribosomal biogenesis factor (*RBIS*)	2652
OAR24_3882451.1	24	3882451	0.125	−0.5533	7.70E-05	Septin 12 (*SEPTIN12*)	2648
s56765.1	12	79260312	0.4917	0.3886	7.87E-05	Protein-tyrosine-phosphatase (*PTPN7*)	175698
s49449.1	8	22519512	0.05	0.7318	9.11E-05	ENSOARG00000027009	523076

Several SNPs mapped near genes with potential biological relevance, including *MICU1* (mitochondrial calcium uptake), *LARP7* (La ribonucleoprotein 7, transcriptional regulator), and *SEPTIN12*. In contrast, OAR20_45605951.1, s72149.1 and s49449.1 were not matched to genes with known function, and they are described as novel, long intergenic non-coding RNA genes (37). Because these genes are poorly characterised, functional interpretation of these SNPs is omitted.

### 3.3 HR-LCMS

Metabolites that were matches to the KEGG *Ovis aries* eicosanoids’ subsection of the arachidonic acid metabolism pathway as described in 3.5.2, are tabled further in Supplementary Tables 1, 2. Prior to treatment, only 5-Oxo-eicosatetraenoic acid and arachidonic acid were matched to detected features. However, these metabolites were not significant within or between groups (*p* > 0.05). In contrast, immediately post-treatment, 5-Oxo-eicosatetraenoic acid, arachidonic acid and PGF_2α_ were matched to features. Of these metabolites, PGF_2α_ was significantly higher in the sponge-treated group compared to the CIDR-treated and control group as per Tukey’s HSD differences per group comparison (Sponge-treated vs. CIDR-treated group difference = 2994.06; Sponge-treated vs. control group difference = 4058.39; *p* < 0.005).

## 4 Discussion

Endocervical secretions, vaginal mucus and immune metabolites contribute to the composition of cervicovaginal fluid (12, 43). It is an important medium through which the ewe, as well as other female livestock, maintain their reproductive tract’s homeostasis by secreting crucial metabolites related to immune function and reproductive cyclicity. As such, this microenvironment must maintain characteristics beneficial to its optimal functioning, such as pH, microbiome composition and viscosity. Contextually, cervicovaginal pH, metabolomic composition, and inflammatory signalling represent complementary but non-equivalent dimensions of the cervicovaginal environment where pH reflects physicochemical conditions, metabolomic profiles integrate host and microbial activity, and eicosanoids such as PGF_2α_ provide specific insight into host inflammatory responses.

The long-term habitation of a foreign object in the vaginal canal likely affects vaginal temperature and aeration, and relatedly, affects the composition of the microbiome. This study supports literature that states the use of intravaginal pessaries alters the vaginal microbiome composition (2, 8, 44) while other authors have hypothesised that pH does not change significantly during CIDR or sponge treatment despite a change in bacterial flora of the vagina in ewes (45, 46). Cervicovaginal fluid varies by species, being neutral in ewes where it ranges from 5.6 to 7.1, compared to ranging from 6.5 to 8.7 in cows (44, 45), which is corroborated by this study’s findings. Nevertheless, the extent to which the observed alterations reflect device-specific effects versus physiological processes inherent to the natural estrous cycle warrants careful consideration.

Studies in livestock have demonstrated that reproductive tract secretions, including cervical mucus, contain lipid mediators associated with inflammatory and endocrine signalling even in the absence of exogenous devices (47). These findings indicate that a basal level of inflammatory and eicosanoid activity is an intrinsic component of the estrous cycle (48). For example, disruption of the ewe’s reproductive tract occurs during her previous parturition if she is multiparous (48). However, the ewes in this study had exceeded the recommended duration of recovery for postpartum recovery and uterine involution if multiparous (16, 17).

PGF_2α_, a downstream metabolite of the KEGG *Ovis aries* arachidonic acid/eicosanoid metabolism pathway, plays a central physiological role in luteolysis and reproductive tract function in ruminants, and its production within uterine and cervical tissues is well established under normal cycling conditions (49–51). However, PGF_2α_ being significantly higher in the Sponge-treated group post-treatment when compared to both the CIDR-treated and control group (*p* < 0.005), combined with significantly elevated cervicovaginal pH in the Sponge-treated group (*p* < 0.05), provides novel support of device-mediated amplification of endogenous immune processes. Contextually, intravaginal sponges have been reported to induce greater local discharge and vaginitis than CIDRs due to their absorptive capacity, triggering intensified local irritation (5). This effect fosters arachidonic acid pathway activation and prostanoid production, which may feasibly result in disruptions of the cervicovaginal environment impactful enough to adversely affect sperm survival (2, 52, 53).

Prostanoid production will be controlled at multiple levels ranging from substrate availability, enzyme activity and receptor abundance, meaning that a SNP could influence PGF_2α_ output in a variety of ways. From the GWAS linear regression model, the strongest signal was OAR25_28537107.1 (OAR25: 28537107 bp), located near *MICU1* showing a notably low *p*-value of 3.66E-07 and represents a rare but high-impact allele capable of shifting pH by more than one unit (although its MAF warrants cautious interpretation as this figure translates to 1.67% of the alleles in the population carrying the minor variant and thus few individuals carrying copies of the minor allele (54)). In contrast, Figure 4’s ten significant SNPs (*p* < 1 × 10^−4^) with moderate to high minor allele frequencies (0.23–0.49) showed consistent and thus biologically plausible effect sizes (*β* = −0.5 to 1.5), suggesting that these loci may contribute genuine variation in pH responses across treatment groups. This is due to moderate minor allele frequencies indicating that these alleles are present in a substantial proportion of the population and their estimated effects are based on observations from many individuals rather than a small number of rare carriers. Thus, their associated effect sizes are more less susceptible to statistical inflation and likely to represent genuine biological contributions to variation in cervicovaginal pH between treatment groups.

Further, principal component analysis showed the absence of major population stratification and confirms that treatment groups differed phenotypically rather than genetically. Accordingly, the QQ plot showed only mild deviation from expectation at the right tail, suggesting that the observed inflation reflects genuine signal and potential associations with cervicovaginal pH rather than systematic bias. The colour gradient of the volcano plot revealed novel additional biological insight by showing that the most significant SNPs tended to have low to moderate MAF, consistent with the notion that less frequent alleles may produce larger phenotypic deviations (54, 55). However, several moderate-frequency alleles (MAF ≈ 0.2–0.4) also produced notable effects, indicating that both rare and common variants may contribute to pH variation.

**Figure 5:**
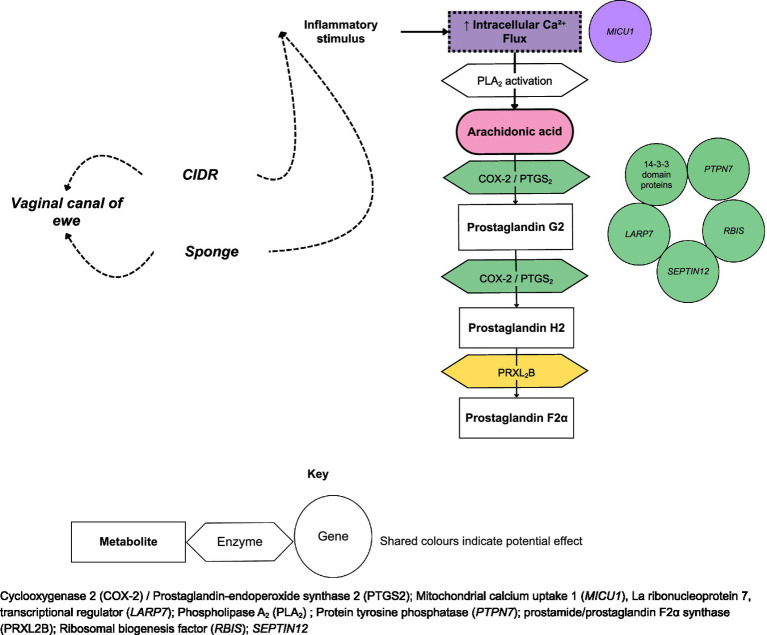
Conceptual model illustrating intravaginal pessary synchronisation method’s interaction with SNP-associated genes to influence the arachidonic acid eicosanoid pathway of Dohne Merino ewes’ cervicovaginal microenvironment post-CIDR, sponge, or control treatment. Adapted from Kanehisa et al. (27).

Several SNPs identified in the GWAS were located near genes involved in mucosal inflammation, epithelial barrier function, mitochondrial stress responses, and transcriptional regulation of which the conceptual model is shown in Figure 5. Arachidonic acid is metabolised by COX enzymes such as prostaglandin endoperoxide synthase 2 (PTGS2/COX-2), thromboxane A synthase, arachidonate 5 lipoxygenase, leukotriene A4 hydrolase and phospholipase A₂ (PLA_2_) (4, 9, 26). This finding is an important mechanistic anchor because PGF_2α_ is a keystone marker of COX-2 activation and mitigation of epithelial irritation within mucosal tissues (56, 57). Thus, although these genes are not classically associated with reproductive physiology, they influence prostaglandin production and reproductive environment homeostasis where genetic variation in these pathways may modulate the vaginal mucosal response to intravaginal devices.

The significance of *MICU1* suggests cellular-level changes in response to treatment. *MICU1* contributes to mitochondrial Ca^2+^ influx and downstream activation of calcium-dependent enzymes, including cPLA₂, which releases arachidonic acid (58). This is because variants in *MICU1* may modify the threshold for mitochondrial Ca^2+^ activation, modifying mucosal oxidative stress, epithelial metabolism, and inflammatory mediator release (59). Thus, enhanced mitochondrial activation under sponge treatment could promote greater arachidonic acid mobilisation and altered epithelial ion transport, indirectly contributing to increases in cervicovaginal pH (60).

14-3-3 domain–containing protein suggests the involvement of intracellular scaffolding proteins that regulate phosphorylation-dependent signalling in this study’s observed results (61, 62). The 14-3-3 family interacts with MAPK, ERK and NF-κB pathways, which all drive COX-2/PTGS2 transcription and influence the inflammation of the vaginal mucosa (63). In ewes exposed to the mechanically harsher sponge-treatment compared to CIDR-treatment, variants that modify 14-3-3 regulatory function may result in stronger activation of stress and inflammatory pathways. This may alter epithelial ion transport and buffering capacity and may contribute to the observed increase in cervicovaginal pH in the sponge-treatment group. Similarly, the protein tyrosine phosphatase gene, *PTPN7*, plays an important role in turning off MAPK-mediated stress signals via dephosphorylation (64). Reduced activity or altered regulation of protein tyrosine phosphatases can thus prolong MAPK and NF-κB activation and increase the transcription of inflammatory mediators like PTGS_2_ (65). Because the sponge device produces greater friction than the CIDR, ewes carrying sensitising alleles at this locus could exhibit exaggerated mucosal inflammatory responses, shifting pH upward through alkalinising inflammatory exudate and epithelial bicarbonate factors and acidifying lactobacilli-derived lactic acid.

*LARP7* modulates the positive transcription elongation factor b (P-TEFb) complex. Increased transcriptional permissiveness that is caused by *LARP7* variation may accelerate expression of stress-responsive and inflammatory genes, including those controlling prostaglandin metabolism and epithelial ion transporters. Thus, under sponge-induced epithelial irritation, this and other similar alleles may potentiate the transcriptional response to mucosal stress. *RBIS* similarly influences ribosomal assembly and global protein synthesis including that of stress-responsive proteins such as COX-2, cytokines, and epithelial ion transporters (66). Ewes with alleles promoting heightened translational capacity may have a more robust inflammatory or reparative response to sponge-induced epithelial irritation. Thus, increased production of alkaline secretions or reduced maintenance of acidic microflora may cause upward shifts in cervicovaginal pH.

*DTNBP1* participates in vesicle trafficking and regulated secretion, and this may influence the release of inflammatory mediators and prostanoids, resulting in altered buffering capacity and thus contributing to elevated vaginal pH (9, 10). *SEPTIN12* is a cytoskeletal protein that also participates in cytokine secretion and is crucial for epithelial barrier integrity (67). Thus, genetic variants affecting septin function could weaken tight junction stability and thereafter allow greater microbial exposure. The sponge device likely increases epithelial stress compared to the CIDR and ewes with reduced barrier robustness may experience amplified innate immune activation.

## 5 Conclusion

The findings of this study should be interpreted within the context of both physiological estrous cycling and device-associated modulation of the cervicovaginal environment. While prostaglandin production and immune activity are fundamental components of normal reproductive physiology in sheep, the observed shifts in cervicovaginal pH and metabolomic profiles indicate that intravaginal devices can alter the magnitude and balance of these processes. This distinction between baseline endocrine-regulated prostaglandin signalling and device-associated amplification is critical for interpreting reproductive outcomes and host responses. Thus, eicosanoid-immunometabotypes of the cervicovaginal fluid of sheep that indicate dysbiosis are characterised by a decrease in cervicovaginal pH and significantly increased activity of the eicosanoid arachidonic acid pathway with PGF_2α_ being a marker metabolite.

The genes linked to significant SNPs may modulate how strongly an animal responds to intravaginal sponge placement, contributing to the higher cervicovaginal pH observed in the sponge-treated group. PGF_2α_ differences should not be interpreted as a cause of higher pH, but rather a biochemical indicator of the epithelial inflammatory response that underlies the observed pH elevation. Together with PCA and QQ plot results showing minimal population stratification, the genomic findings support a biologically consistent model where intravaginal pessaries used for estrus synchronisation increase mucosal inflammation and through mediation by prostaglandin pathways, interacts with host genetics to produce the observed phenotypic differences. Importantly, the GWAS findings indicate that host genetic variation in pathways governing calcium flux, inflammatory signalling, and transcriptional control may determine the extent to which physiological prostaglandin responses are amplified in the presence of intravaginal devices, providing a mechanistic basis for inter-individual variability in cervicovaginal pH and metabolomic outcomes.

Changes in cervicovaginal pH may be influenced by factors such as diet. These alternative factors, including parity, were accounted for in the experimental design and statistical analysis when selecting systemic markers. Thus, the concept of eicosanoid-immunometabotypes has potential translational relevance to other phenotypes in livestock and other mammals and represents an understanding of eicosanoid metabolism in livestock that allows the design of effective detection and intervention strategies to improve livestock production and welfare.

## Data Availability

The original contributions presented in the study are publicly available. This data can be found here: https://www.ebi.ac.uk/metabolights/MTBLS14823. Genotypic data can be found here: https://doi.org/10.5281/zenodo.20375099.

## References

[1] Pluta K Jones PRH Drabińska N Ratcliffe N Carrington SD Lonergan P . The potential of volatile organic compound analysis in cervicovaginal mucus to predict estrus and ovulation in estrus-synchronized heifers. J Dairy Sci. (2021) 104:1087–98. doi: 10.3168/jds.2020-19024, 33189280

[2] Abril-Parreño L Meade KG Krogenæs AK Druart X Fair S Cormican P. Conserved and breed-specific differences in the cervical transcriptome of sheep with divergent fertility at the follicular phase of a natural oestrus cycle. BMC Genomics. (2021) 22:752. doi: 10.1186/s12864-021-08060-9, 34666676 PMC8527727

[3] Romero G Restrepo I Muelas R Bueso-Ródenas J Roca A Díaz JR. Within-day variation and effect of acute stress on plasma and milk cortisol in lactating goats. J Dairy Sci. (2015) 98:832–9. doi: 10.3168/jds.2014-8052, 25497807

[4] Chhonker YS Bala V Murry DJ. Quantification of eicosanoids and their metabolites in biological matrices: a review. Bioanalysis. (2018) 10:2027–46. doi: 10.4155/bio-2018-0173, 30412686 PMC6562698

[5] Oliveira JK Martins G Esteves LV Penna B Hamond C Fonseca JF . Changes in the vaginal flora of goats following a short-term protocol of oestrus induction and synchronisation with intravaginal sponges as well as their antimicrobial sensitivity. Small Rumin Res. (2013) 113:162–6. doi: 10.1016/j.smallrumres.2013.02.011

[6] Reinoso-Peláez EL Saura M González-Recio Ó González C Fernández A Peiro-Pastor R . Impact of oestrus synchronization devices on ewes vaginal microbiota and artificial insemination outcome. Front Microbiol. (2023) 14:1063807. doi: 10.3389/fmicb.2023.1063807, 37032869 PMC10076614

[7] Manes J Hozbor F Alberio R Ungerfeld R. Intravaginal placebo sponges affect negatively the conception rate in sheep. Small Rumin Res. (2014) 120:108–11. doi: 10.1016/j.smallrumres.2014.05.006

[8] Bartlewski PM Seaton P Szpila P Oliveira MEF Murawski M Schwarz T . Comparison of the effects of pretreatment with Veramix sponge (medroxyprogesterone acetate) or CIDR (natural progesterone) in combination with an injection of estradiol-17β on ovarian activity, endocrine profiles, and embryo yields in cyclic ewes superovulated in the multiple-dose Folltropin-V (porcine FSH) regimen. Theriogenology. (2015) 84:1225–37. doi: 10.1016/j.theriogenology.2015.07.002 26231309

[9] Calder PC. “Eicosanoids“. In: Harwood J Lloyd-Evans E, editors. Essays in Biochemistry, vol. 64 (2020). p. 423–41. 32808658 10.1042/EBC20190083

[10] Ricciotti E FitzGerald GA. Prostaglandins and inflammation. Arterioscler Thromb Vasc Biol. (2011) 31:986–1000. doi: 10.1161/ATVBAHA.110.207449, 21508345 PMC3081099

[11] Duckett SK Thomas AR Udoka A Greene MA. 213 nutritional and genetic influences on fatty acid composition of beef and lamb. J Anim Sci. (2021) 99:111–1. doi: 10.1093/jas/skab235.202

[12] Wu S Hugerth LW Schuppe-Koistinen I Du J. The right bug in the right place: opportunities for bacterial vaginosis treatment. NPJ Biofilms Microbiomes. (2022) 8:34. doi: 10.1038/s41522-022-00295-y, 35501321 PMC9061781

[13] Goot H. Effect of light on spring breeding of mutton merino ewes. J Agric Sci. (1969) 73:177–80. doi: 10.1017/S0021859600014350

[14] Ramírez Ramírez AI Delgado Tiburcio GI Cruz-Espinoza F Herrera-Corredor AC. Photoperiod and its relationship to sheep reproduction. Agro Productividad. (2021). doi: 10.32854/agrop.v14i10.1620

[15] Department of Agriculture, Land Reform and Rural Development R of SA. Abstract of Agricultural Statistics. Pretoria: Directorate Statistics and Economic Analysis (2025).

[16] Pesántez-Pacheco JL Heras-Molina A Torres-Rovira L Sanz-Fernández MV García-Contreras C Vázquez-Gómez M . Maternal metabolic demands caused by pregnancy and lactation: association with productivity and offspring phenotype in high-yielding dairy ewes. Animals. (2019) 9:295. doi: 10.3390/ani9060295, 31151216 PMC6617180

[17] Evkuran Dal G Enginler SO Cetin AC Baykal K Sabuncu A. B-mode and Doppler ultrasonographic assessment of uterine involution in ewes treated with two different doses of ProstaglandinF2α. Acta Sci Vet. (2020) 48. doi: 10.22456/1679-9216.105041

[18] Mohan K Kumar N. Comparative evaluation of estrus synchronization protocols on reproductive performance and estrus behavior in Barbados black belly sheep. Vet World. (2023) 16:2244–9. doi: 10.14202/vetworld.2023.2244-2249 38152269 PMC10750750

[19] Quinlan J Myburgh SJ de Vos D. The hydrogen-ion concentration of the vaginal secretion of merino sheep during oestrus, dioestrus, and pregnancy, with some remarks on its influence on sex-determination, and the influence of the vaginal temperature at the time of mating on conception. Onderstepoort J Vet Res. (1941) 17:105–14.

[20] Sitaresmi PI Wulandari A Hudaya MF Santoso S Herdis H Anwar RI . Estrus detection on different thoroughbred crossbreed horses with vaginal smear and vaginal acidity method. IOP Conf Ser: Earth Environ Sci. (2024) 1360:012021. doi: 10.1088/1755-1315/1360/1/012021

[21] Waters Inc. UNIFI Scientific Information System. Milford: (2024).

[22] Sun J Xia Y. Pretreating and normalizing metabolomics data for statistical analysis. Genes Dis. (2024) 11:100979. doi: 10.1016/j.gendis.2023.04.018, 38299197 PMC10827599

[23] Posit Team. RStudio: Integrated Development for R [Windows]. Boston: Posit Software, PBC; (2025). Available online at: http://posit.co/

[24] Pang Z Lu Y Zhou G Hui F Xu L Viau C . MetaboAnalyst 6.0: towards a unified platform for metabolomics data processing, analysis and interpretation. Nucleic Acids Res. (2024) 52:W398–406. doi: 10.1093/nar/gkae253, 38587201 PMC11223798

[25] Ritchie ME Phipson B Wu D Hu Y Law CW Shi W . Limma powers differential expression analyses for RNA-sequencing and microarray studies. Nucleic Acids Res. (2015) 43:e47–7. doi: 10.1093/nar/gkv007, 25605792 PMC4402510

[26] Zheng WB Zou Y Elsheikha HM Liu GH Hu MH Wang SL . Serum metabolomic alterations in beagle dogs experimentally infected with Toxocara canis. Parasit Vectors. (2019) 12:447. doi: 10.1186/s13071-019-3703-5, 31506092 PMC6737696

[27] Kanehisa M Furumichi M Sato Y Matsuura Y Ishiguro-Watanabe M. KEGG: biological systems database as a model of the real world. Nucleic Acids Res. (2025) 53:D672–7. doi: 10.1093/nar/gkae909, 39417505 PMC11701520

[28] Le Faouder P Baillif V Spreadbury I Motta JP Rousset P Chêne G . LC–MS/MS method for rapid and concomitant quantification of pro-inflammatory and pro-resolving polyunsaturated fatty acid metabolites. J Chromatogr B Analyt Technol Biomed Life Sci. (2013) 932:123–33. doi: 10.1016/j.jchromb.2013.06.014, 23831705

[29] Lee J Mok H Lee DY Park S Ban M Choi J . UPLC-MS/MS-based profiling of eicosanoids in RAW264.7 cells treated with lipopolysaccharide. Int J Mol Sci. (2016) 17:508. doi: 10.3390/ijms17040508, 27058537 PMC4848964

[30] Schmidt L Garscha U. Species-specific optimization of oxylipin ionization in LC–MS: a design of experiments approach to improve sensitivity. Anal Bioanal Chem. (2025) 417:1807–18. doi: 10.1007/s00216-025-05759-6, 39891659 PMC11913994

[31] Yuan ZX Majchrzak-Hong S Keyes GS Iadarola MJ Mannes AJ Ramsden CE. Lipidomic profiling of targeted oxylipins with ultra-performance liquid chromatography-tandem mass spectrometry. Anal Bioanal Chem. (2018) 410:6009–29. doi: 10.1007/s00216-018-1222-4, 30074088 PMC6314687

[32] R Core Team. R: A Language and Environment for Statistical Computing. Vienna: R Foundation for Statistical Computing; (2024). Available online at: https://www.R-project.org/.

[33] Purcell S Chang C PLINK Version 1.9. (2015). Available online at: www.cog-genomics.org/plink/1.9/.

[34] Durinck S Moreau Y Kasprzyk A Davis S Moor BD Brazma A . BioMart and Bioconductor: a powerful link between biological databases and microarray data analysis. Bioinformatics. (2005) 21:3439–40. doi: 10.1093/bioinformatics/bti525, 16082012

[35] Lawrence M Gentleman R Carey V. Rtracklayer: an R package for interfacing with genome browsers. Bioinformatics. (2009) 25:1841–2. doi: 10.1093/bioinformatics/btp328, 19468054 PMC2705236

[36] Lawrence M Huber W Pagès H Aboyoun P Carlson M Gentleman R . Software for computing and annotating genomic ranges. PLoS Comput Biol. (2013) 9:e1003118. doi: 10.1371/journal.pcbi.1003118, 23950696 PMC3738458

[37] Warnes G Gorjanc G Leisch F Man M. Genetics: Population Genetics. (2021). Available online at: https://CRAN.R-project.org/package=genetics.

[38] Dyer SC Austine-Orimoloye O Azov AG Barba M Barnes I Barrera-Enriquez VP . Ensembl 2025. Nucleic Acids Res. (2025) 53:D948–57. doi: 10.1093/nar/gkae1071, 39656687 PMC11701638

[39] Fadista J Manning AK Florez JC Groop L. The (in)famous GWAS P-value threshold revisited and updated for low-frequency variants. Eur J Hum Genet. (2016) 24:1202–5. doi: 10.1038/ejhg.2015.269, 26733288 PMC4970684

[40] Guo Y Huang Y Hou L Ma J Chen C Ai H . Genome-wide detection of genetic markers associated with growth and fatness in four pig populations using four approaches. Genet Sel Evol. (2017) 49:21. doi: 10.1186/s12711-017-0295-4, 28196480 PMC5307927

[41] Devlin B Roeder K. Genomic control for association studies. Biometrics. (1999) 55:997–1004. doi: 10.1111/j.0006-341X.1999.00997.x, 11315092

[42] Hayes BJ Bowman PJ Chamberlain AJ Goddard ME. Invited review: genomic selection in dairy cattle: Progress and challenges. J Dairy Sci. (2009) 92:433–43. doi: 10.3168/jds.2008-1646, 19164653

[43] Yang J Lee SH Goddard ME Visscher PM. GCTA: a tool for genome-wide complex trait analysis. Am J Hum Genet. (2011) 88:76–82. doi: 10.1016/j.ajhg.2010.11.011, 21167468 PMC3014363

[44] Tsiligianni T Karagiannidis A Brikas P Saratsis P. Chemical properties of bovine cervical mucus during normal estrus and estrus induced by progesterone and/or PGF2α. Theriogenology. (2001) 56:41–50. doi: 10.1016/S0093-691X(01)00541-6, 11467517

[45] Swartz JD Lachman M Westveer K O’Neill T Geary T Kott RW . Characterization of the vaginal microbiota of ewes and cows reveals a unique microbiota with low levels of lactobacilli and near-neutral pH. Front Vet Sci. (2014) 1:19. doi: 10.3389/fvets.2014.00019 26664918 PMC4672155

[46] Manes J Fiorentino MA Kaiser G Hozbor F Alberio R Sanchez E . Changes in the aerobic vaginal flora after treatment with different intravaginal devices in ewes. Small Rumin Res. (2010) 94:201–4. doi: 10.1016/j.smallrumres.2010.07.021, 38826717

[47] Martinez-Ros P Gonzalez-Bulnes A Garcia-Rosello E Rios-Abellan A Astiz S. Effects of short-term intravaginal progestagen treatment on fertility and prolificacy after natural breeding in sheep at different reproductive seasons. J Appl Anim Res. (2019) 47:201–5. doi: 10.1080/09712119.2019.1599899

[48] Senger PL. Pathways to Pregnancy and Parturition. Second Revised ed. Washington, United States of America: Cadmus Professional Communications (2003).

[49] Esposito G Irons PC Webb EC Chapwanya A. Interactions between negative energy balance, metabolic diseases, uterine health and immune response in transition dairy cows. Anim Reprod Sci. (2014) 144:60–71. doi: 10.1016/j.anireprosci.2013.11.007, 24378117

[50] McCracken JA Custer EE Lamsa JC. Luteolysis: a neuroendocrine-mediated event. Physiol Rev. (1999) 79:263–323. doi: 10.1152/physrev.1999.79.2.263, 10221982

[51] Silvia WJ Lewis GS Mccracken JA Thatcher WW Wilson L. Hormonal regulation of uterine secretion of prostaglandin F2α during luteolysis in ruminants1. Biol Reprod. (1991) 45:655–63. doi: 10.1095/biolreprod45.5.655 1756203

[52] Laguardia-Nascimento M Branco KMGR Gasparini MR Giannattasio-Ferraz S Leite LR Araujo FMG . Vaginal microbiome characterization of Nellore cattle using metagenomic analysis. PLoS One. (2015) 10:e0143294. doi: 10.1371/journal.pone.0143294, 26599789 PMC4657983

[53] Martinez-Ros P Lozano M Hernández F Tirado A Rios-Abellán A López-Mendoza MC . Intravaginal device-type and treatment-length for ovine estrus synchronization modify vaginal mucus and microbiota and affect fertility. Animals. (2018) 8:226. doi: 10.3390/ani8120226, 30501021 PMC6316288

[54] Bokulich NA Łaniewski P Adamov A Chase DM Caporaso JG Herbst-Kralovetz MM. Multi-omics data integration reveals metabolome as the top predictor of the cervicovaginal microenvironment. PLoS Comput Biol. (2022) 18:e1009876. doi: 10.1371/journal.pcbi.1009876, 35196323 PMC8901057

[55] Park JH Gail MH Weinberg CR Carroll RJ Chung CC Wang Z . Distribution of allele frequencies and effect sizes and their interrelationships for common genetic susceptibility variants. Proc Natl Acad Sci USA. (2011) 108:18026–31. doi: 10.1073/pnas.1114759108, 22003128 PMC3207674

[56] Manolio TA Collins FS Cox NJ Goldstein DB Hindorff LA Hunter DJ . Finding the missing heritability of complex diseases. Nature. (2009) 461:747–53. doi: 10.1038/nature08494, 19812666 PMC2831613

[57] Morita I. Distinct functions of COX-1 and COX-2. Prostaglandins Other Lipid Mediat. (2002) 68-69:165–75. doi: 10.1016/S0090-6980(02)00029-1, 12432916

[58] Watanabe K. Prostaglandin F synthase. Prostaglandins Other Lipid Mediat. (2002) 68–69:401–7. doi: 10.1016/S0090-6980(02)00044-8 12432932

[59] Tomar D Thomas M Garbincius JF Kolmetzky DW Salik O Jadiya P . MICU1 regulates mitochondrial cristae structure and function independently of the mitochondrial Ca^2+^ uniporter channel. Sci Signal. (2023) 16:eabi8948. doi: 10.1126/scisignal.abi8948, 37098122 PMC10388395

[60] Gijón MA Leslie CC. Regulation of arachidonic acid release and cytosolic phospholipase A_2_. J Biol Chem. (1999) 274:12512–8. doi: 10.1074/jbc.274.14.12512 10080535

[61] Schwecht I Nazli A Gill B Kaushic C. Lactic acid enhances vaginal epithelial barrier integrity and ameliorates inflammatory effects of dysbiotic short chain fatty acids and HIV-1. Sci Rep. (2023) 13:20065. doi: 10.1038/s41598-023-47172-y, 37973920 PMC10654711

[62] Huang DW Sherman BT Lempicki RA. Systematic and integrative analysis of large gene lists using DAVID bioinformatics resources. Nat Protoc. (2009) 4:44–57. doi: 10.1038/nprot.2008.211, 19131956

[63] Sherman BT Hao M Qiu J Jiao X Baseler MW Lane HC . DAVID: a web server for functional enrichment analysis and functional annotation of gene lists (2021 update). Nucleic Acids Res. (2022) 50:W216–21. doi: 10.1093/nar/gkac194, 35325185 PMC9252805

[64] Munier CC Ottmann C Perry MWD. 14-3-3 modulation of the inflammatory response. Pharmacol Res. (2021) 163:105236. doi: 10.1016/j.phrs.2020.105236, 33053447

[65] Martín-Vázquez E Cobo-Vuilleumier N López-Noriega L Lorenzo PI Gauthier BR. The PTGS2/COX2-PGE_2_ signaling cascade in inflammation: pro or anti? A case study with type 1 diabetes mellitus. Int J Biol Sci. (2023) 19:4157–65. doi: 10.7150/ijbs.86492, 37705740 PMC10496497

[66] Kaltschmidt B Kaltschmidt C Baeuerle PA. Transcription factor NF-κB is activated by intracellular hydrogen peroxide in neuronal cells. J Biol Chem. (2002) 277:18229–34. doi: 10.1074/jbc.M20081520011889120

[67] Poligone B Baldwin AS. Positive and negative regulation of NF-κB by COX-2. J Biol Chem. (2001) 276:38658–64. doi: 10.1074/jbc.M106599200 11509575

[68] Pochard C Gonzales J Bessard A Mahe MM Bourreille A Cenac N . PGI2 inhibits intestinal epithelial permeability and apoptosis to alleviate colitis. Cell Mol Gastroenterol Hepatol. (2021) 12:1037–60. doi: 10.1016/j.jcmgh.2021.05.001, 33971327 PMC8342971

[69] Hu F Wang L Wang Z Liang J Li J Mao G . Re-characterization of some factors influencing aerosol sampling in the workplace: results from field studies. J Occup Health. (2014) 56:351–8. doi: 10.1539/joh.13-0221-OA, 25069894

[70] Martínez-Sena T Luongo G Sanjuan-Herráez D Castell JV Vento M Quintás G . Monitoring of system conditioning after blank injections in untargeted UPLC-MS metabolomic analysis. Sci Rep. (2019) 9:9822. doi: 10.1038/s41598-019-46371-w, 31285473 PMC6614502

