# Supplementary material for: Eicosanoid-immunometabotypes reveal genotypic links to cervicovaginal pH in progestagen synchronised South African Dohne Merino ewes
Source: Front Vet Sci. 2026 Jul 21;13:1851840. doi: 10.3389/fvets.2026.1851840 (PMC13435568; doi:10.3389/fvets.2026.1851840)
Supplement: Supplementary file 1 [file Table_1.DOCX]

Supplementary Material

# 1. Supplementary Materials and Methods

### 1.1. Cervicovaginal pH

To determine cervicovaginal pH, the FLOQSwabs were dipped in 10 mL isotonic solution (0.9% w/v NaCl) and allowed to soak for 45 minutes, then vortexed and transferred to glass tubes of 20 mL. The glass electrode was cleaned and ensured to be free of contaminants by rinsing with distilled water. The pH meter was calibrated with buffer of pH 4, 7 and 10 and must have passed calibration before sample measurement. Stabilisation of the reading was waited for ca. 3 minutes and after stabilisation, two measurements were taken for accuracy, where both pH values were recorded.

### 1.2. Metabolite extraction and instrument method

Two mL of 50:50 methanol (MeOH) and water were pipetted directly into the vial, after which the vial was vortexed at maximum speed for 30 seconds and transferred to a 25 mL volumetric flask. This step was repeated twice more while transferring the reconstituted internal standard to the 25 mL volumetric flash each time. The same 50:50 MeOH:Water mix was used to fill the volumetric flask to the final 25 mL volume, closed, and gently inverted to mix. The lowest target analyte concentration in the mix was 2 ng/mL and the highest 3000 ng/mL. The standards’ solution mix was injected for LC-HRMS analysis. All solvents used, including acetonitrile (ACN), MeOH, and water, were sourced from Romil (Romil-UpS™, Microsep, South Africa).

After collection of cervicovaginal samples, the swab tip was placed in an Eppendorf safe-lock tube (1.5 mL) and 1 mL of acetonitrile was pipetted into the Eppendorf tube. The tube was placed in a foam tube holder and sonicated for 5 minutes. The Eppendorf tube was opened and the swab tip removed using a tweezer rinsed in acetonitrile, closed and centrifuged at 5 minutes. The Eppendorf tube was then opened and 0.9 mL of supernatant pipetted into a glass vial (5 mL). The glass vial was placed to dry under a gentle stream of nitrogen overnight without a lid. The glass vial was closed with a lid and stored in a refrigerator until analysis. During reconstitution, 0.1 mL of acetonitrile and 0.1 mL of water were added to the glass vial. The vial was closed and centrifuged for 30 seconds, thereafter, submitted for LC-HRMS analysis.

Compound separation and detection were performed using a Waters^®^ Synapt G2 high definition mass spectrometry (HDMS) system (Waters Inc., Milford, Massachusetts, USA). The system comprises of a Waters Acquity Ultra Performance Liquid Chromatography (UPLC^®^) system hyphenated to a quadrupole-time-of-flight (QTOF) instrument. The system was operated with MassLynx™ (version 4.1) software (Waters Inc., Milford, Massachusetts, USA) for data acquisition and processing. The source conditions were as follows: the capillary voltage for ESI was 2.6 kV and 2.0 kV for positive and negative mode ionisation. The source temperature was set at 120 ºC, the sampling cone voltage at 25 V, extraction cone voltage at 4.0 V and cone gas (nitrogen) flow at 10.0 L/Hr. The desolvation temperature was set at 350 ºC with a gas (nitrogen) flow of 600.0 L/Hr.

Quantitative data-independent acquisition (DIA) was done using two simultaneous acquisition functions with low and high collision energy (MS^E^ approach) with a QTOF instrument. Fragmentation was performed using high-energy collision-induced dissociation (CID). The fragmentation energy was set at 2 V and 3 V for the trap and collision energy, respectively. The ramping was set from 3 to 4 V and 20 to 40 V for the trap and transfer collision energy, respectively. Mass spectral scans were collected every 0.3 seconds. The raw data was collected in the form of a continuous profile. Mass to charge ratios (*m/z*) between 50 and 1 200 Da were recorded.

Separation was completed using a reverse phase step gradient elution scheme from 97% H2O (0.1% formic acid) to 100% acetonitrile (0.1% formic acid). The gradient started with an isocratic flow (hold 0.1 min) followed by a linear increase to 100% ACN; subsequently the column was washed for 1 min followed by conditioning and re-establishing of initial conditions to allow for equilibration before the start of the next run for the complete elution scheme. The column temperature was kept constant at 40 ºC and the flow rate was set at 0.4 mL/min for the entire run giving a total run time of 20 min. Injection volumes were set at 5 µL. A Kinetex® 1.7 µm EVO C18 100 Å (2.1 mm ID x 100 mm length) column was used. The positive and negative ion mass spectra were collected in separate chromatographic runs (employing the same separation conditions).

### 1.3. Quality control of samples

The field blank was taken by removing the sterile swab from its sterile holder, and gently waving the swab near the ewes and along the longitude of the restraining pen at each sampling point to determine if the observed results reflect actual differences between treatment groups or if they are influenced by environmental contamination (1). Additionally, features of the solvent blank were determined by running the solvents used in analysis through the same sample processing procedure used for swabs, without any sample present. This allowed the detection of impurities or contaminants that may have been introduced by solvents or reagents (2). Apparatus may also be a cause of background noise and contamination thus swab blank features were obtained by processing a sterile swab in the laboratory as a biological sample. The features found in swab blanks were subsequently excluded in reporting.

### 1.4. Target metabolites

The following metabolites are listed in the KEGG *Ovis aries* database as eicosanoid metabolites under the arachidonic acid metabolism pathway metabolites (3): Arachidonic acid, prostaglandin E_2_ (PGE_2_), prostaglandin D_2_ (PGD_2_), prostaglandin F2 α (PGF2a), 6-keto-Prostaglandin F1 α (6-keto-PGF1a), thromboxane B_2_ (TXB_2_), leukotriene B4 (LTB_4_), leukotriene C_4_ (LTC_4_), leukotriene D4 (LTD4), leukotriene E4 (LTE4), 5-hydroxyeicosatetraenoic acid (5-HETE), 5-oxo-eicosatetraenoic acid (5-oxo-ETE), 12-hydroxyeicosatetraenoic acid (12-HETE), 15-Hydroxyeicosatetraenoic acid (15-HETE), lipoxin A4, lipoxin B4, 5,6-epoxyeicosatrienoic acid (5,6-EET), 8,9-epoxyeicosatrienoic acid (8,9-EET), 11,12-epoxyeicosatrienoic acid (11,12-EET), 14,15-epoxyeicosatrienoic acid (14,15-EET), 5,6-dihydroxyeicosatrienoic acid (5,6-DHET), 8,9-dihydroxyeicosatrienoic acid (8,9-DHET), 11,12-dihydroxyeicosatrienoic acid (11,12-DHET), 14,15-dihydroxyeicosatrienoic acid (14,15-DHET) and 20-hydroxyeicosatetraenoic acid (20-HETE).

# References

1.	Hu F, Wang L, Wang Z, Liang J, Li J, Mao G, et al. Re‐characterization of Some Factors Influencing Aerosol Sampling in the Workplace: Results from Field Studies. Journal of Occupational Health. 2014 Sep;56(5):351–8. doi:10.1539/joh.13-0221-OA

2.	Martínez-Sena T, Luongo G, Sanjuan-Herráez D, Castell JV, Vento M, Quintás G, et al. Monitoring of system conditioning after blank injections in untargeted UPLC-MS metabolomic analysis. Sci Rep. 2019 Jul 8;9(1):9822. doi:10.1038/s41598-019-46371-w

3.	Kanehisa M, Furumichi M, Sato Y, Matsuura Y, Ishiguro-Watanabe M. KEGG: biological systems database as a model of the real world. Nucleic Acids Research. 2025 Jan 6;53(D1):D672–7. doi:10.1093/nar/gkae909
